# An exploratory cross-sectional study on Mental health literacy of Spanish adolescents

**DOI:** 10.1186/s12889-024-18933-9

**Published:** 2024-05-31

**Authors:** Clara González-Sanguino, Jairo Rodríguez-Medina, Jesús Redondo-Pacheco, Elena Betegón, Lorena Valdivieso-León, María Jesús Irurtia

**Affiliations:** 1 Department of Psychology, University of Valladolid, Valladolid, Spain; 2 Department of Pedagogy, University of Valladolid, Valladolid, Spain; 3 Department of Personality, Evaluation and Psychological Treatment, University of Salamanca, Salamanca, Spain

**Keywords:** Adolescents, Discrimination, Mental health, Mental health literacy, Stigma

## Abstract

**Background:**

Mental health literacy (MHL) is especially important for young people, but comprehensive studies on MHL in adolescents are limited, with no nationwide studies in Spain. This research aims to study MHL among Spanish adolescents and its relationship with sociodemographic factors.

**Methods:**

An exploratory study is carried out using stratified random sampling in Spanish adolescents (*N* = 1000), aged 12–16 years and balanced in terms of gender, age and territorial distribution. Data collection took place in October and November 2023 through online surveys using the CAWI methodology. Sociodemographic variables, contact with mental health and the Spanish version of the Mental Health Literacy Questionnaire (MHLq-E), a self-administered instrument of 32 Likert-type items (1–5) that assesses the dimensions of help-seeking skills, knowledge about causes and symptoms, and stigma, were evaluated. Descriptive and multivariate analyses of variance (MANOVA) were conducted.

**Results:**

In general, adequate levels of literacy were observed, although some aspects related to help-seeking towards teachers, stigmatising attitudes towards people of low economic status and knowledge of severe mental health problems could be improved. The results show contact with previous mental health problems as a key variable for stigma and knowledge about symptomatology together with age. Likewise, gender and family educational level were found to be related to the ability to seek professional help.

**Conclusion:**

This study provides information on levels of MHL among Spanish adolescents and highlights significant socio-demographic variables. These findings pave the way for interventions aimed at improving adolescents' understanding, attitudes and skills to manage mental health problems, making possible to adapt content and focus on specific groups, thus increasing its effectiveness.

**Supplementary Information:**

The online version contains supplementary material available at 10.1186/s12889-024-18933-9.

## Background

Mental health literacy (MHL) is defined as "the body of knowledge and beliefs about mental disorders that assist in their recognition, management or prevention" ( [1], p.19). MHL emphasizes those dimensions that can improve mental health outcomes [2], including information on how to acquire and maintain good mental health, understanding mental disorders and their treatments, decreasing stigma against physical illness (PI), and ultimately increasing the effectiveness of help-seeking [2, 3].

Although the concept of MHL is important throughout society, young people are particularly relevant, revealing themselves as a group in which mental health problems (MHPs) are especially prevalent. More than 13% of adolescents suffer from some mental health problem, with an estimated one suicide occurring every 11 min [4], with suicidal ideation and its association with anxiety and depression among young people being particularly alarming [5, 6]. Therefore, MHL among adolescents is essential in the prevention of MHPs, as well as to promote early detection and reduce stigma.

Existing studies suggest that some specific variables may influence MHL levels, such as sex and previous contact with people who have MHPs. With regard to sex, results from different studies suggest that young women have higher levels of MHL [7, 8], presenting more positive attitudes and fewer stereotypes in relation to people with mental disorders, compared to young men [9, 10]. In addition, different research reports higher levels of MHL in people who report knowing someone with a mental health problem [11, 12]. This prior contact seems to increase the impact of MHL promotion interventions among young people [13].

For adolescents, studies on MHL are smaller compared to the adult population [10, 14], and among the different studies in the last decade, most were conducted with older adolescents [15]. Thus, Radez et al. [16] found that socioeconomic disadvantage and low levels of educational attainment were not associated with MHL, and women and people with MHPs were more likely to seek help from different sources [9]. In addition, being female, older and having a higher level of education were found to be related to the ability to recognize MHPs [17, 18].

In Spain, research with adolescents has focused on specific components of the construct, such as stigma or attitudes, as in the validation of the CAMI scale in adolescents [19], where stigmatizing attitudes were found in relation to authoritarianism, as well as better attitudes in girls. Other investigations are conducted from an MHL perspective, although none of them provide a global measure of the construct and its different dimensions. First, it is necessary to mention the implementation of an online program "EspaiJove.net" [20], from which they developed the EMHL [21], an instrument that assesses adolescents' MHL regarding MHPs. Their results show that adolescents have medium–high levels of recognition of MHPs, but low levels of knowledge about them. In addition, they find that help-seeking is generally medium–high in relation to friends, parents and professionals, but that young people would not seek help from their teacher. Furthermore, the levels of stigma found were medium, with no improvement in any of the conditions after the intervention. Another study is from García-Soriano and Roncero [22] focused on the MHL of obsessive–compulsive disorder (OCD), in which they found that most adolescents did recognize the interference of OCD and would recommend a formal source of help, although they confused it with schizophrenia or depression, and there was stigma surrounding the perception that OCD is dangerous.

Considering the above, the aim of the present research is to study MHL and its various dimensions among adolescents in a sample of the Spanish population, to determine what they know about mental health, their attitudes or stigma and what help-seeking skills they have, as well as to identify differences in the distinct dimensions and their relationship with various socio-demographic variables.

## Methods

### Sampling and sample characteristics

A total of 1000 Spanish adolescents were sampled using stratified random sampling (sampling error 3.1% with a confidence level of 95.5% for an infinite universe and under the assumption of maximum indeterminacy). Participants were aged 12–16 years (M = 14; SD = 1.41) with sex, age and distribution in the territory balanced groups.

As main characteristics, families reported a medium socioeconomic level (more than 80% have low or medium monthly family income), and medium–high educational level (slightly more than half of the sample had university studies), with most of the adolescents attending public schools and only 7.3% having a diagnosis of MHPs, PI or recognized disability. On the other hand, most of the participating adolescents considered themselves to be of Western European ethnicity and more than 60% said they did not know anyone with MHPs, with only 4.3% claiming to suffer from them or to have experienced them at first hand. In Table 1 the sociodemographic characteristics of the sample can be seen in detail.

**Table 1:** Socio-demographic characteristics and contact with mental health problems in the sample

Variable	N	%
**Gender**		
Male	493	49.3
Female	500	50
None of the above	7	0.7
**Age**		
12	199	19.9
13	199	19.9
14	203	20.3
15	200	20
16	199	19.9
**Place of residence size**		
0–10.000	231	23.1
10.001–50.000	255	25.5
50.001–200.000	226	22.6
Over 200.000	288	28.8
**Monthly family income**^a^		
Up to 2160	419	41.9
Between 2160–4000	474	47.4
Over 4000	107	10.7
**Family education level**		
No schooling	3	0.3
Primary	39	3.9
Obligatory secondary education/Vocational training	382	38.2
University	445	44.5
Master’s degree/PhD	131	13.1
**Ethnic group**		
Western Europe/North America	928	92.8
Others	72	7.2
**Type of school**		
Public	695	69.5
Subsidized private	256	25.6
Private	49	4.9
**Mental Health Problems / Physical illness /Disability**		
Yes	73	7.3
No	927	92.7
**Mental Health Contact**		
Don't know anyone	623	62.3
Family member or friend	328	32.8
MHP in self	49	4.9

^a^The monthly family income has been determined based on the minimum wage in Spain

### Procedure

The research is part of the project "Stigma and discrimination as a factor of vulnerability in childhood" funded by the La Caixa Child Vulnerability Flash Call (FS23-IB). Sampling and data collection were carried out in October and November 2023. Adults with children aged 12–16 were contacted using a random sampling methodology with paired replacement. The Spanish panel is composed of 105,002 persons (according to ISO20252 standard). The sample was stratified according to sex, age and Nielsen area of residence. Data collection was carried out by the company “Analysis and Research” through Computer Assistance Web Interview (CAWI) methodology. Families interested in participating were first provided with information about the study and informed consent. Then, they answered some socio-demographic questions (5 min), instructing them to leave their child alone to answer the rest of the survey (25 min). All data collected was anonymous (researchers were never able to access the identification of any of the participants) and approved by the ethical committee of the University of Valladolid (PI 23-3245NOHCUV).

### Variables and instruments

#### Sociodemographics

Evaluated by means of ad hoc questions. Questions answered by the parents: age of the son or daughter; place of residence; monthly family income (calculated based on the minimum wage for two persons); highest educational level of the family; type of school the child attends; and presence of MHPs, physical illness or disability. Questions answered by adolescents: gender; ethnic group; contact with MHPs.

#### Mental health literacy

It was assessed using the Mental Health Literacy questionnaire—young people (MHLq) [11] in its Spanish version (MHLq-E) [23]. Consisting of 32 items (Table S1, supplementary material), participants are asked to express their degree of agreement with each of the statements on a five-point Likert scale (1 = strongly disagree; 2 = disagree; 3 = neither agree nor disagree; 4 = agree; 5 = strongly agree). The instrument is composed of 4 subscales: (1) Help-seeking and skills, referring to adolescents' strategies and ability to seek help for a mental health problem. Made up of 9 items. E.g. *If a friend had a mental health problem I would talk to their parents or encourage them to go to a psychologist*; (2) Stigma, attitudes and prejudices towards mental health. Made up of 5 items E.g. *Depression is not a real mental disorder*; (3) Knowledge regarding causes, knowledge about the causes of mental problems. Made up of 8 items. E.g. *Physical exercise helps improve mental health, or talking about problems helps improve mental health*; (4) Knowledge regarding symptoms, characteristics of mental problems. Made up of 10 items. E.g. *People with schizophrenia usually have delusions*. In all subscales, a higher score reflects a higher MHL, which means more knowledge about the causes and symptomatology of the disorders, better help-seeking skills, as well as better attitudes or less stigmatization. The total mental health literacy score is calculated by adding the total score of the items. The instrument has shown sufficient evidence of both reliability and validity of the interpretation of its scores [11] and has been used in the evaluation of the construct in the implementation of intervention programs [24].

### Data analysis

Descriptive analyses were carried out on the sociodemographic characteristics of the sample and in relation to the MHLq-E results. No missing values were found and the presence of multivariate outliers was checked using Mahalanobis D^2^ distances. Then, a series of multivariate analysis of variance (MANOVA) was performed to identify relationships between the dependent variables and the factors considered. Each of the dimensions of the instrument was considered as a dependent variable: (1) Help-seeking and skills; (2) Stigma; (3) Knowledge regarding causes; and (4) Knowledge regarding symptoms. The independent variables were gender, age, family education level, ethnic group and previous contact with MHPs. Due to the poor representativeness of some groups, and the impossibility of carrying out analyses with them, certain variables were re-categorized: gender, individuals corresponding to a non-binary gender were eliminated (*N* = 7), leaving two categories: female and male; family education level: basic/secondary vs. university; ethnic group: Western Europe/North America vs. other; mental health contact: don't know anyone vs. knowing someone.

M Box tests were carried out to calculate equality between variance–covariance matrices, as well as Levene's test for equality, using the Pillai trace statistic and Bonferroni post hoc tests. The full-scale internal consistency was checked using the Cronbach's alpha coefficients for ordinal data [25] and McDonald's omega [26] and subsequently the reliability of each of the subscales was checked.

## Results

### Overall results in mental health literacy

The overall scores reveal MHL levels above the average of the questionnaire, replicated in all sub-dimensions of the MHL. On a scale of 0 to 100 for total MHL scores, participants obtained a mean score of 76.59 (SD = 9.57). The highest score was in Knowledge about Causes (M = 79.61, SD = 11.63) and the lowest in Knowledge about Symptoms (M = 74.31, SD = 11.25). The mean scores in Help Seeking and Stigma were 76.51 (SD = 12.65) and 75.91 (SD = 17.19), respectively.

However, when looking at individual items, some aspects need to be highlighted. For example, in relation to the Help-seeking factor, more than 90% of the adolescents revealed that their help mechanisms would be peer support, family and seeking medical attention (items 1, 5 and 19), although in less than 45% of cases, they would turn to a friend's parents if they had an MHPs, or to a teacher (items 8 and 29). With respect to the Stigma dimension, the affirmative responses were not so unanimous, with more than 20% of adolescents believing that people with MHPs come from families with little money, and more than 30% believing that depression is not a real mental disorder (items 12 and 26).

In terms of knowledge about the causes of MHPs, it's worth noting that more than 94% of adolescents were aware of the advantages of early identification and treatment of MHPs, and almost 92% were aware of the link between MHPs and drug use (items 16 and 27). Regarding the dimension of knowledge about symptoms, the results are more disparate, finding that certain symptomatology linked to MHPs is well known, more than 85% know that a person with depression feels unhappy, and more than 85% know that a person with anxiety can feel panic in feared situations, (items 3 and 11), although only 70% know that a person with schizophrenia has delusions (item 31), and less than 44% recognize the duration of symptoms as important when it comes to establishing a MHPs (item 25).

Additionally, excellent reliability values were obtained for the full scale (α = 0.92 [0.92, 0.93], and ωt = 0.94 [0.92, 0.93]) and adequate for the four subscales that make up the instrument (α and ωt > 0.8). The mean scores on the total scale and sub-dimensions, as well as the response frequencies for each item can be seen in detail in Table 2 and in Table S1 at the supplementary material.

**Table 2:** Descriptive of Mental Health Literacy questionnaire—young people in Spanish (MHLq-E)

	**MHLq-** **Help-seeking**		**MHLq-** **Stigma**		**MHLq-Causes**		**MHLq-Symptoms**		**MHLq-TOTAL**	
**Variable**	**M**	**SD**	**M**	**SD**	**M**	**SD**	**M**	**SD**	**M**	**SD**
**Gender**										
Female	78.21	12.26	76.56	17.57	79.86	11.89	74.63	11.34	77.31	9.89
Male	74.85	12.83	75.16	16.77	79.44	11.28	73.96	11.17	75.85	9.16
**Age**										
12	75.61	13.52	73.21	17.04	78.11	12.71	73.09	12.33	75.01	9.97
13	75.67	12.37	74.79	17.89	79.54	11.22	73.44	11.91	75.86	9.86
14	76.31	12.61	74.63	17.12	79.73	12.60	73.53	10.62	76.05	9.75
15	76.97	12.57	78.70	16.20	80.39	11.34	75.23	11.09	77.82	9.63
16	77.97	12.09	78.21	17.15	80.28	10.00	76.28	9.91	78.19	8.26
**Place of residence size**										
0–10.000	77.44	11.88	75.52	15.95	79.33	11.12	74.36	10.19	76.66	9.30
10.001–50.000	76.27	13.02	76.24	15.89	79.52	12.38	73.91	11.93	76.49	9.80
50.001–200.000	75.91	13.72	76.50	17.71	79.47	12.32	74.05	11.54	76.48	10.29
Over 200.000	76.44	12.05	75.47	18.84	80.02	10.79	74.84	11.24	76.69	9.04
Monthly income*										
Up to 2160	77.14	13.00	74.34	18.18	79.75	11.89	74.68	11.94	76.48	10.07
Between 2160–4000	76.71	12.12	76.78	16.62	79.87	11.44	74.09	10.52	76.86	9.12
Over 4000	73.13	13.16	78.18	15.22	77.92	11.36	73.83	11.60	75.77	9.63
**Family education level**										
Basic/secondary	78.05	13.04	75.15	12.05	79.47	12.05	74.74	11.63	76.85	10.09
University	75.38	12.25	76.47	11.31	79.71	11.31	74.00	10.96	76.39	9.18
**Ethnic group**										
Western Europe/ North America	76.22	12.60	76.21	16.70	79.46	11.43	74.11	11.13	76.50	9.51
Others	79.84	12.81	72.53	21.85	81.29	13.63	76.64	12.35	77.57	10.32
**Type of school**										
Public	76.64	12.78	75.12	17.83	79.55	12.06	74.22	11.35	76.38	9.75
Subsidized private	76.41	12.10	77.73	14.81	79.50	10.70	73.96	10.83	76.90	9.03
Private	75.17	13.71	77.55	19.04	80.93	9.93	77.45	11.65	77.78	9.80
**Mental Health Problems / Physical illness /Disability**										
Yes	76.43	12.70	75.78	17.06	79.53	11.73	74.04	11.17	76.45	9.61
No	77.47	12.08	77.53	18.78	80.65	10.15	77.74	11.68	78.35	9.04
**Mental Health Contact**										
No	76.71	12.76	74.39	17.60	79.11	11.76	73.39	11.57	75.90	9.86
Yes	76.17	12.48	78.42	16.20	80.43	11.37	75.83	10.54	77.71	8.98
**TOTAL**	76.51	12.65	75.91	17.19	79.61	11.63	74.31	11.25	76.59	9.57

*M* mean, *SD* Standard Deviation

*The monthly family income has been determined based on the minimum wage in Spain; Mental Health Contact: No = not knowing someone with mental health problems, Yes = knowing someone with mental health problems (friend, familiar, oneself)

### Interaction effects between variables

**Fig. 1:**
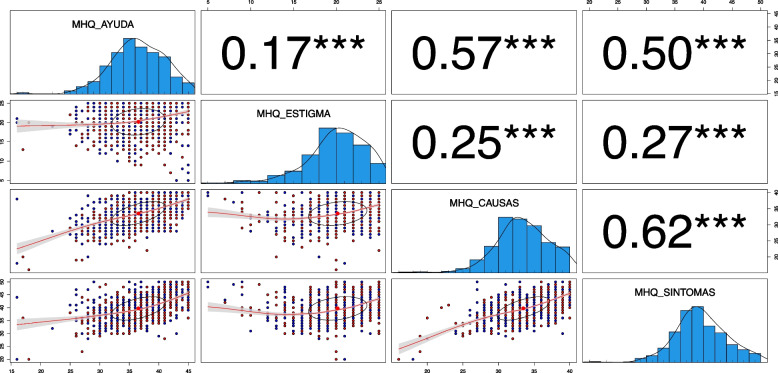
Distributions and Correlations among the Mental Health Literacy dimensions. Note. MHQ_HELP = Help-seeking and skills; MHQ_STIGMA = Stigma; MHQ_CAUSES = Knowledge regarding Causes; MHQ_SYMPTOMS = Knowledge regarding Symptoms

The absence of multicollinearity is indicated by Pearson's correlation values of less than 0.9, ranging between 0.17 and 0.62 (Fig. 1). Multivariate outliers were then analyzed using Mahalanobis D^2^ distances. Eighteen multivariate outliers were identified as significant at the α = 0.001 confidence level [27]. The maximum D^2^ value was 44.56. These cases were studied in order to identify any common patterns or characteristics (e.g. being part of a particular group, sharing some quality of sociodemographic variables). Since no pattern was found, these cases were eliminated from further analysis.

A series of univariate analyses of variance were then conducted to test for the presence of interaction effects between each pair of independent variables for each of the dependent variables. No significant interaction effects were observed between gender and family education level for any of the dependent variables (Help-seeking F(1,989) = 0.57, *p* = 0.45; Stigma F(1,989) = 0.88, *p *= 0.34; Knowledge about Causes F(1,989) = 1.72, *p* = 0.19, Knowledge about Symptoms F(1,989) = 0.28, *p* = 0.59). However, main effects on the variable Help were observed for both gender (F(1,989) = 17.71, *p* < 0.01) and educational level (F(1,989) = 10.22, *p* < 0.01). There was also no significant interaction between gender and age (Help-seeking F(1,989) = 1.41, *p* = 0.31; Stigma F(1,989) = 0.60, *p* = 0.44; Knowledge about Causes F(1,989) = 0.16, *p* = 0.90, Knowledge about Symptoms F(1,989) = 0.46, *p* = 0.50). However, main effects of the Age factor were observed for the variables Help-seeking (F(1,989) = 4.97, *p* < 0.05), Stigma (F(1,989) = 13.23, *p* < 0.01), Knowledge about Causes (F(1,989) = 4.44, *p* < 0.05) and Knowledge about Symptoms (F(1,989) = 10.60, *p* < 0.01).

Main effects of the Ethnic Group (dichotomous) factor were also observed for the variables Help-seeking (F(1,989) = 7.48, *p* < 0.01) and Knowledge about Symptoms (F(1,989) = 4.26, *p* < 0.05). Again, no interaction effects were observed between the Gender and Ethnic Group (dichotomous) factors.

Similarly, with respect to the Prior Contact factor, main effects were observed for the variables Stigma (F(1,989) = 2.23, *p* < 0.01) and Knowledge about Symptoms (F(1,989) = 12.88, *p* < 0.01). There were also no interaction effects between gender and previous contact. Finally, no significant interaction effects were observed between the factors Age, Educational Level, Ethnic Group (dichotomous) or Prior Contact on any of the dependent variables.

### Study of multidimensional differences

A series of multivariate analyses of variance were carried out with each of the factors as independent variables and the dependent variables Help-seeking, Stigma, Knowledge about Causes and Symptoms. Due to the number of factors (5) included in the analysis and the high number of interactions between them (26) an alpha level = 0.001 was established. Table 3 shows the main results of this analysis.

**Table 3:** Results of Results of multivariate comparisons of the means (MANOVA)

Effect	df_1_	df_2_	Λ	F	p	ηp^2^
Age	4	958	.979	4.91	< .001	0.02
Gender	4	958	.975	6.00	< .001	0.02
Family education Level	4	958	.977	5.53	< .001	0.02
Ethnic group	4	958	.985	3.41	= .008	0.01
Mental Health Contact	4	958	.978	6.48	< .001	0.03

*df*  degrees of freedom, Λ = Wilks’s lambda, ηp^2^ = eta partial squared

First, no interaction effects were observed between the factors Gender, Age, Educational Level, Ethnic Group and Prior Contact. Statistically significant differences in Mental health literacy were observed as a function of Age (V = 0.02, F (4, 958) = 4.91, *p* < 0.001, ηp^2^ = 0.019), Gender (V = 0.02, F (4, 958) = 6.00, *p* < 0.001, η^2^ = 0.019), Educational level (V = 0.02, F (4, 958) = 5.53, *p* < 0.001, ηp^2^ = 0.019), and Prior Contact (V = 0.02, F (4, 958) = 5.15, *p* < 0.001, ηp^2^ = 0.019). No statistically significant effects were found for the Ethnic Group factor.

Subsequently, following the approach proposed by Stevens [28], a series of MANOVA's were performed for each pair of groups generated by the factors that were found to be significant. Women obtained higher mean scores than men on all variables. However, statistically significant differences were only found in the variable Help-seeking (F(1,991) = 17.83, *p* < 0.001, η^2^ = 0.018) with a small effect size. There was also a significant effect of Education Level found in the variable Help-seeking (F(1,991) = 10.05, *p* < 0.005, η^2^ = 0.018) with a small effect size. In this variable, families with secondary education or less obtained a higher mean score (M = 78.05, SD = 13.04) than families with higher education (M = 75.38, SD = 12.25). Regarding the Age factor, subsequent analyses showed significant differences between the 12 and 16—year-old groups (F(4, 391) = 3.44, *p* < 0.001, η^2^ = 0.03) exclusively on the variable Stigma (F(1, 397) = 8.13, *p* < 0.005, η^2^ = 0.02). The factor Previous Contact with MHPs also produced a significant effect (F(4, 958) = 6.48, *p* < 0.001, ηp^2^ = 0.03), specifically, participants with previous contact with people with MHPs obtained higher mean scores than those without previous contact in the variables Stigma (F(1, 991) = 12.32, *p* < 0.001, η^2^ = 0.012) and Knowledge about symptoms (F(1, 991) = 10.40, *p* < 0.005, η^2^ = 0.01). Tables S2-S5 show the subsequent analyses performed and can be found in the supplementary material.

## Discussion

The present research provides an analysis of the general levels of Mental Health Literacy and its different dimensions, as well as key socio-demographic variables in a sample of Spanish adolescents. In general terms, MHL is above the average of the questionnaire. This implies that most of the adolescents responded to the questions showing appropriate knowledge about mental health, as well as good help-seeking skills and attitudes towards disorders. However, there are aspects of the MHL dimensions that reveal shortcomings that could be improved.

Adolescents show adequate resources to ask for help, although the lack of trust in teachers stands out, as well as the fact that they would not warn their friends' parents if they had psychological problems. This may be due to not being labelled as "informers" and the lack of knowledge of families, although it may also reflect a certain lack of trust in adults. On the other hand, the lack of confidence in teachers is striking and worrying, as they should be a clear agent of help in these cases, as well as a key vehicle for detection due to the access and amount of time they spend with pupils. Other studies also replicate not seeing teachers as a source of help [21, 29] consistent with those found by Radez et al. [16] in their systematic review, in which they report that in general young people prefer to talk about their MHPs with peers. Additionally, in another review [30], it is noted that adolescents' negative beliefs about mental health and lack of trust in professionals is one of the main barriers for seeking professional help, while for parents, structural barriers like trust with gatekeepers, i.e. teachers, was fundamental in the process of asking for help. Further training of teachers in mental health is needed, so that they can identify and address early needs in relation to MHPs, being seen as competent agents to make the necessary referrals to health services.

In relation to stigma, although the overall scores do not reveal high levels of stigma, there are still certain prejudices among adolescents, such as the belief that depression is not really a mental disorder, that mental disorders occur in people "with little money" or that mental disorders do not affect feelings. These stigmatizing beliefs often lead to discrimination against peers with MHPs [31, 32] as well as are accompanied by feelings of shame and guilt [33], and can negatively affect professional help-seeking [16, 30]. Fighting stigma in relation to mental health has been made a priority for the World Health Organization [34], so correcting the above stereotypes and dysfunctional beliefs about certain disorders should be a line of action in adolescents.

In addition, the symptom knowledge dimension was the lowest of the entire scale, with a considerable number of adolescents having trouble recognizing the relevance of the duration of MHPs (over 60%), and symptomatology of certain disorders (e.g., over 20% had trouble identifying symptoms of schizophrenia or anxiety). Ignorance of schizophrenia has been shown in other study [35], where also around 30% of their sample of adolescents did not know how to identify the disorder. In addition, some problems are also found in the causes, such as the lack of connection with a good diet (more than 40% did not recognize this) and healthy lifestyle habits (almost 25% did not identify alcohol consumption as a MHPs cause).

Regarding the socio-demographic characteristics of the adolescents, differences were found according to age and previous contact with MHPs. Specifically, less stigma was found in older adolescents and those with more contact with MHPs, the latter also showing more significate knowledge about symptomatology. Thus, contact seems to emerge as a key variable highlighting the importance of knowing others with MHPs and disclosure strategies, being also the variable with the largest effect size. Having prior contact with MHPs increases awareness of the symptomatology and also reduces stigma. These results are consistent with other studies, which have shown the importance of knowing examples of MHPs in real and positive cases, through models or relevant people who tell how the symptomatology is, being effective intervention strategies and breaking stereotypes [14, 36, 37]. In addition, greater contact has also been shown to be relevant in other studies to be associated with better help-seeking skills [11, 12]. On the other hand, younger adolescents are the group with more stigma, establishing themselves as a more vulnerable target group for interventions, with other studies showing that younger adolescents also have poorer help-seeking skills [16, 30, 38, 39].

On the other hand, another important variable in relation to MHL is gender, finding that female adolescent had a greater predisposition and skills to ask for help. This is consistent with other studies, which also find that girls had higher MHL [37], as well as, in general, females tend to ask for help more than males [7, 8]. The effect of gender on MHL could be attributed to the way symptoms of mental disorders are identified and perceived in both genders [39], and the assumption of masculine stereotypes such as "boys don’t cry" that negatively affect men [40]. Furthermore, families with a higher level of education also stand out, where less help was also found compared to those with only primary or basic education. These results disagree with previous literature [41, 42], although they should be interpreted with caution, as in other studies education has been considered a reflection of socio-economic status, whereas in this research education simply reflects studies undertaken, which if unrelated to mental health are not necessarily related to MHL. In either case, educational level in parents is identified as an important variable, as they are the main figures who will role model and help build knowledge and skills in adolescents [43].

The strengths of this research include the size of the sample and the novelty of the data studied, which allow us to learn more about adolescent MHL, an area where more knowledge is needed to prevent and address MHPs. However, the present study also has some limitations. In relation to the type of sampling, the lack of access to populations with low socio-economic status as well as to rural populations or minority ethnic groups stands out. On the other hand, although the sample size (1000 participants) is considerable, there are limitations in terms of representativeness in some variables, such as the lack of adolescents of non-binary gender, or with MHPs, where such small groups have not allowed specific analyses to be carried out on them. This is probably due to the type of sampling and survey methodology used, which did not allow for the inclusion of these groups. Future studies on samples with greater representativeness of these groups, or carried out specifically on these populations, would be necessary. On the other hand, despite the measures taken to guarantee the quality of the responses (survey company with ISO 20252 quality standards), the validity of the responses of the adolescents could be affected by the context in which they are answered and the nature of the questions with high social desirability. For example, instructions to parents explicitly stated that adolescents should answer the questionnaire alone, but this could not be controlled, so perhaps some adolescents might feel embarrassed if their parents met them when answering, and might feel social desirability in certain questions, such as those related to discrimination or stigma.

## Conclusion

This research provides data on the MHL of Spanish adolescents, as well as identifies key socio-demographic variables to be able to intervene and improve adolescents' knowledge, attitudes and skills to cope with MHPs. Certain areas of concern are identified regarding adolescent’s MHL: improving knowledge about certain disorders, enhancing certain prejudicial attitudes and promote skills to ask for professional help in school settings. As recommendations for future research and intervention guidelines, it is considered necessary to design and implement programs to promote MHL in Spain at a national level. At this moment, different countries such Canada [44], Australia [45], United Kingdom [46], and Japan [47] have initiated educational courses related to MHL nationwide. The present study shows the key variables on which to intervene at a socio-demographic level to favor the effectiveness of these programs, such as the youngest, adolescent males and those who have had no previous contact with MHPs. In addition, to improve mental health literacy among gatekeepers such as teachers or parents is recommended.

### Supplementary Information

Supplementary Material 1.

## Data Availability

Sampling and data collection were carried out in October and November 2023. The datasets used in this study are available from the research team coordinator (CGS: clara.gonzalez.sanguino@uva.es) upon reasonable request.

## Abbreviations

MHL: Mental health literacy
PI: Physical illness
MHPs: Mental health problems
OCD: Obsessive-compulsive disorder
MHLq-E: Mental Health Literacy questionnaire - young people in Spanish
MANOVA: Multivariate analysis of variance
