# Supplementary material for: An exploratory cross-sectional study on Mental health literacy of Spanish adolescents
Source: BMC Public Health. 2024 May 31;24:1469. doi: 10.1186/s12889-024-18933-9 (PMC11143666; doi:10.1186/s12889-024-18933-9)
Supplement: Supplementary file 1 — Supplementary Material 1. [file 12889_2024_18933_MOESM1_ESM.pdf]

Supplementary Material

Table 4. Items of the MHLq-E and results obtained.

Item	Factor	Description	% 1 to 2	% 4 to 5
MHQ1	Help-Seeking and skills	If a friend of mine developed a mental disorder, I would offer my support	0.6	94.3
MHQ16	Knowledge Causes	The earlier mental disorders are identified and treated, the better	1	94.2
MHQ27	Knowledge Causes	Drug addiction can cause mental disorders	0.9	92.7
MHQ19	Help-Seeking and skills	If a friend of mine developed a mental disorder, I would encourage them to seek medical help	1.2	91.6
MHQ5	Help-Seeking and skills	If I myself had a mental health problem, I would seek help from my family	2.3	91.2
MHQ13	Help-Seeking and skills	If a friend of mine developed a mental disorder, I would listen to them without judging or criticizing	1.6	88.7
MHQ17r	Stigma	Only adults have mental disorders	4.7	88.5
MHQ30	Knowledge Causes	Doing enjoyable activities helps improve mental health	1.4	88.5
MHQ28	Knowledge Symptoms	Mental disorders affect people's thoughts	1.3	87.9
MHQ6	Help-Seeking and skills	If a friend of mine developed a mental disorder, I would encourage them to see a psychologist	2	87.3
MHQ9	Knowledge Causes	Sleeping well helps improve mental health	2	86.9
MHQ3	Knowledge Symptoms	A person with depression feels very unhappy	2.8	86.3
MHQ11	Knowledge Symptoms	A person with anxiety can feel panic in situations they fear	0.9	86.2
MHQ2	Knowledge Causes	Physical exercise helps improve mental health	2.4	86
MHQ32	Knowledge Causes	Talking about problems with someone helps improve mental health	1.6	85.1
MHQ10	Help-Seeking and skills	If I myself had a mental disorder, I would seek professional help (psychiatrist or psychologist)	2.1	83.3
MHQ22	Knowledge Symptoms	One of the symptoms of depression is losing interest or stopping enjoying most things	2.5	83.3
MQH7r	Stigma	Mental disorders do not affect people's behavior	9.6	79.2
MHQ33	Knowledge Symptoms	Highly stressful situations can cause mental disorders.	2.7	79
MHQ12r	Stigma	People with mental disorders come from families with little money	5	78.9
MHQ31	Knowledge Symptoms	A person with schizophrenia may see and hear things that no one else sees or hears	2.3	75.6
MHQ14	Knowledge Causes	Alcohol consumption can cause mental disorders	4.7	75.4
MHQ20	Help-Seeking and skills	If I myself had a mental disorder, I would seek help from my friends	6.8	73.4
MHQ15r	Stigma	Mental disorders do not affect people's feelings	11.6	72.9
MHQ4	Knowledge Symptoms	People with schizophrenia usually have delusions (e.g., they may believe they are constantly being watched or persecuted)	1.9	70
MHQ23	Knowledge Symptoms	A person with anxiety avoids situations that they find stressful	5.1	68.3

MHQ26r	Stigma	Depression is not a real mental disorder	14.2	67.8
MHQ18	Knowledge Symptoms	Brain malfunction can cause the onset of a mental disorder	4.1	66.8
MHQ21	Knowledge Causes	Following a balanced diet helps improve mental health	6.1	59.8
MHQ29	Help-Seeking and skills	If a friend of mine developed a mental disorder, I would tell a teacher or tutor	18.5	45.1
MHQ25	Knowledge Symptoms	The duration of symptoms is an important aspect in determining whether a person has a mental disorder or not	7.7	43.8
MHQ8	Help-Seeking and skills	If a friend of mine developed a mental disorder, I would talk to their parents	18	43

Note. R = reversed items

Table 5. Results of a One-Way MANOVA and Univariate Effects for Gender

	Value	F	df_1	df_2	p	ηp^2
Pillai's trace	.024	5.99	4	988	< .001	.02
Wilk's lambda	.976	5.99	4	988	< .001	.02
Hotelling's trace	.024	5.99	4	988	< .001	.02

	Gender						
	Female (n = 500)		Male (n = 493)				
**DV**	***M***	***SD***	***M***	***SD***	***F***	***p***	η^2
Mental Health							
Help-seeking	37.16	4.42	35.95	4.62	17.83	< .001	.018
Stigma	20.31	3.52	20.03	3.36	1.64	= .20	
Causes	33.56	3.81	33.42	3.61	0.334	= .56	
Symptoms	39.85	4.54	39.59	4.47	0.865	= .35	

Note. M = mean; SD = Standard Deviation; df = degrees of freedom; ηp2 = eta partial squared.

Table 6. Results of a One-Way MANOVA for Age

	V	F	df1	df2	p	ηp^2
12 vs 13	.007	0.694	4	391	.596	
12 vs 14	.008	0.776	4	391	.541	
12 vs 15	.030	3.029	4	394	.017	.03
12 vs 16	.034	3.438	4	391	< .001	.03
13 vs 14	.001	0.111	4	392	.978	
13 vs 15	.018	1.804	4	392	.127	
13 vs 16	.029	2.903	4	389	.02	.03
14 vs 15	.018	1.859	4	395	.117	
14 vs 16	.028	2.892	4	392	.022	.03
15 vs 16	.005	0.547	4	392	.681	

Note. V = Pillai's trace; df = degrees of freedom; ηp2 = eta partial squared

Table 7. Results of a One-Way MANOVA and Univariate Effects for Education Level

	Value	F	df1	df2	p	ηp^2
Pillai's trace	.021	5.28	4	988	< .001	.02
Wilk's lambda	.976	5.28	4	988	< .001	.02
Hotelling's trace	.021	5.28	4	988	< .001	.02

	Education Level						
	Sec / Inf (n = 422)		Uni / Sup (n = 571)				
**DV**	**M**	**SD**	**M**	**SD**	**F**	**p**	**η^2**
Mental Health							
Help-seeking	37.09	4.70	36.16	4.41	10.05	< .005	.018
Stigma	20.01	3.74	20.29	3.19	1.57	= .21	
Causes	33.42	3.85	33.54	3.60	0.28	= .59	
Symptoms	39.90	4.66	39.59	4.38	1.18	= .28	

Note. M = mean; SD = Standard Deviation; V = Pillai’s trace; df = degrees of freedom; ηp2 = eta partial squared.

Table 8. Results of a One-Way MANOVA and Univariate Effects for Mental Health Contact

	Value	F	df1	df2	p	ηp²
Pillai's trace	.026	6.48	4	988	< .001	.03
Wilk's lambda	.974	6.48	4	988	< .001	.03
Hotelling's trace	.026	6.48	4	988	< .001	.03

	**Previous Contact**						
	**No (n = 619)**		**Yes (n = 374)**				
**DV**	**M**	**SD**	**M**	**SD**	**F**	**p**	**η^2**
Mental Health							
Help-seeking	36.63	4.59	36.43	4.51	0.44	= .51	
Stigma	19.88	3.52	20.66	3.24	12.32	< .001	.012
Causes	33.33	3.74	33.75	3.65	2.91	= .08	
Symptoms	39.36	4.64	40.31	4.21	10.40	< .005	.01

Note. M = mean; SD = Standard Deviation; V = Pillai's trace; df = degrees of freedom; ηp2 = eta partial squared.
